# Societal Cost of Ischemic Stroke in Romania: Results from a Retrospective County-Level Study

**DOI:** 10.3390/brainsci11060689

**Published:** 2021-05-24

**Authors:** Stefan Strilciuc, Diana Alecsandra Grad, Vlad Mixich, Adina Stan, Anca Dana Buzoianu, Cristian Vladescu, Mihaela Adela Vintan

**Affiliations:** 1 Department of Neurosciences, “Iuliu Hatieganu” University of Medicine and Pharmacy, 4000012 Cluj-Napoca, Cluj, Romania; adinadora@yahoo.com (A.S.); adelavintan@yahoo.com (M.A.V.); 2 RoNeuro Institute for Neurological Research and Diagnostic, No. 37 Mircea Eliade Street, 400354 Cluj-Napoca, Cluj, Romania; diana.grad@ssnn.ro; 3 Department of Public Health, Babes-Bolyai University, No. 7 Pandurilor Street, 400376 Cluj-Napoca, Cluj, Romania; mixich@gmail.com; 4 Department of Pharmacology, Toxicology and Clinical Pharmacology, “Iuliu Hatieganu” University of Medicine and Pharmacy, 400012 Cluj-Napoca, Cluj, Romania; abuzoianu@umfcluj.ro; 5 National School of Public Health Management and Professional Development, No 31 Vaselor, Street, 030167 Bucharest, Romania; cristian.vladescu@gmail.com; 6 Department of Public Health, University of Medicine and Pharmacy Victor Babes, No.2 Eftimie Murgu Square, 300041 Timisoara, Timis, Romania

**Keywords:** cost of stroke, societal perspective, first-ever stroke

## Abstract

Background: Health policies in transitioning health systems are rarely informed by the economic burden of disease due to scanty access to data. This study aimed to estimate direct and indirect costs for first-ever acute ischemic stroke (AIS) during the first year for patients residing in Cluj, Romania, and hospitalized in 2019 at the County Emergency Hospital (CEH). Methods: The study was conducted using a mixed, retrospective costing methodology from a societal perspective to measure the cost of first-ever AIS in the first year after onset. Patient pathways for AIS were reconstructed to aid in mapping inpatient and outpatient cost items. We used anonymized administrative and clinical data at the hospital level and publicly available databases. Results: The average cost per patient in the first year after stroke onset was RON 25,297.83 (EUR 5226.82), out of which 80.87% were direct costs. The total cost in Cluj, Romania in 2019 was RON 17,455,502.7 (EUR 3,606,505.8). Conclusions: Our costing exercise uncovered shortcomings of stroke management in Romania, particularly related to acute care and neurorehabilitation service provision. Romania spends significantly less on healthcare than other countries (5.5% of GDP vs. 9.8% European Union average), exposing stroke survivors to a disproportionately high risk for preventable and treatable post-stroke disability.

## 1. Introduction

Based on the latest WHO projections, stroke will continue to be a severe challenge to population health, as it is expected to maintain its leading position as a global cause of mortality until 2060 [1]. Studies have shown that post-stroke patients experience a wide range of adverse outcomes, such as aphasia [2], post-stroke anxiety [3], and depression [4], among others. 

Although the majority of cases are reported in the population over 50 years of age [5], numbers among the younger population have been rising as well [6,7]. Out of the three main stroke categories, ischemic stroke is the most prevalent [8,9,10]. Procedures such as intravenous thrombolysis and mechanical thrombectomy have been used in stroke-like units in Romania to improve post-stroke outcomes for eligible patients [11], decreasing costs [12,13].

With 18.5% of the residing population being over 65 years old [14] and a high incidence of stroke-related risk factors such as obesity, smoking, and lack of exercise [15], the general Romanian population is at risk of an increasing number of cases of non-communicable diseases, such as ischemic stroke. 

In-depth data on stroke cases are scarce in Romania. A total of 959,319 stroke-related deaths were observed between 1994–2017, of which over half (54.3%) were women [16]. Until now, two costs-of-illness studies focusing on in-hospital costs generated by stroke patients admitted in Romanian public hospitals have been published [17,18]. 

With a growing number of thrombolysis centers, an ongoing pandemic, and a low percentage of GDP allocated to the health sector [19], the cost of illness studies targeting leading non-communicable diseases such as stroke are imperious to finance this sector according to its needs and to generate evidence for future policy changes. Our study is the first cost-of-stroke study to focus exclusively on first-ever ischemic stroke patients treated in a Romanian public hospital and appraise stroke-related costs incurred at a societal level. We aim to analyze direct and indirect costs from a societal perspective in the first year after stroke onset.

## 2. Materials and Methods

This cost-of-illness study was performed using a retrospective research design based on secondary data. For our costing model, we analyzed an anonymized database provided by the Department of Neurosciences of Iuliu Hațieganu University of Medicine and Pharmacy, supplemented by data from various public sources. We employed a mixed methodology (bottom-up [20], using the costs generated by acute ischemic stroke patients within the Cluj County Emergency Hospital (CEH); and top-down [20], which takes budgets allocated to CEH for specific procedures such as thrombolysis, and other public data on chronic care from the framework contract, on pensions and informal care [11,21,22]). This study was designed from the societal perspective [23] to include multiple types of costs incurred by ischemic stroke, impacting society as a whole. The study’s main objective was to estimate in-hospital direct costs and indirect costs of first-ever acute ischemic stroke patients from Cluj county who have been treated in a stroke-like center (CEH) between the 1 January 2019 and 31 December 2019.

### 2.1. AIS Case Definition

Our in-hospital cohort of analysis was formed based on the following inclusion criteria: first-ever case of acute ischemic stroke confirmed by a CT or MRI, having a primary diagnosis or one of the first three secondary diagnoses coded with I63 according to ICD-10, age over 18, residency in Cluj county, and hospitalized at CEH between 1 of January and 31 of December 2019 at two neurological wards (Neurology 1 and Neurology 2). Patients were excluded based on age (minors), past or present diagnosis of hemorrhagic stroke or transient ischemic attack (TIA), known history of stroke, and receiving treatment in Cluj but residing in another county. For patients included in our study cohort, we excluded hospitalization caused by stroke relapse and have included only AIS index cases. Based on the costs generated by first-ever AIS patients, we calculated averages, which served as a base for estimating the direct costs.

We conducted semi-structured interviews with various key informants from within the healthcare system at the local and national levels using an interview guide tailored based on the stakeholder’s field of expertise to identify stroke patient pathways, clinical protocols, and hospital administrative coding practices. We used a database containing anonymized diagnostic information from CEH and NRITIS (provided by the Department of Neurosciences) to identify the study population, based on the International Classification of Diseases, 10th revision (ICD-10). 

First, acute ischemic stroke cases were identified as patients with a primary diagnosis of I63 (Cerebral infarction) regardless of secondary diagnoses. Based on informant consultations, we also included patients with a primary diagnosis of G81 (hemiplegia and hemiparesis) with I63 recorded as any of the first three secondary diagnoses during 2019. According to our interviews with key stakeholders, AIS patients are routinely coded with G81 to increase per-patient tariffs. Based on a subgroup of AIS patients already matched upon receiving the database, 17.10% of patients with an index case received have been eligible for thrombolysis and have a primary diagnosis coded with G81 followed by secondary diagnosis coded with I63. The practice of purposely miscoding acute ischemic stroke patients is used in order to increase hospital funding. Identifying a first-ever ischemic stroke was challenging due to the fragmentation of electronic medical health records at the national level. Medical histories could not be correlated across several hospital databases due to data anonymity and lack of access. Patients previously treated for AIS at CEH were excluded based on medical history. CEH medical records require physicians to describe patient status and diagnosis. Based on this string field, we performed several operations for case selection using the wildcard filter option of Tableau Prep data cleaning software. Keywords used to identify candidates for the manual exclusion of patients with prior stroke were “recurrent”, “repeated”, “repetition”, “relapse”, and “antecedent”. We also excluded deliberate (e.g., I63 classification otherwise labeled explicitly as TIA) and accidental (e.g., I63 classification described explicitly as HS) miscoding using the same method, with appropriate synonyms for each criterion.

### 2.2. Patient Pathway

In Romania, patients diagnosed with AIS are treated in the acute phase using a “Priority Action” protocol, which focuses on the patient’s pathway from stroke onset to their medical care received in the emergency unit and transfers to the neurology ward [11]. As for the patients’ treatment during their hospitalization on the neurology and neurorehabilitation wards, physicians use unstandardized approaches informed by recommendations from international clinical guidelines [24,25,26]. To fill information gaps regarding the AIS patient pathway in Romania, we have conducted semi-structured interviews tailored to the stakeholder’s expertise. Based on those semi-structured interviews, we have identified: (1) cost categories, (2) public and private healthcare providers, and (3) in- and out-of-hospital trajectory for patients treated in neurology wards. In 2019, in Romania, 43 centers provided thrombolytic therapy [27], out of which four also provided mechanical thrombectomy (Priority Action for Acute Strokes, 2019). 

### 2.3. Data Sources

For this retrospective costing study, we used an anonymized database provided by the Department of Neurosciences of the Iuliu Hatieganu University of Medicine and Pharmacy containing data from the CEH administrative database and the National Registry for Interventional Treatment in Ischemic Stroke (NRITIS). The resulting database includes information on AIS patients’ socio-demographic characteristics (age, gender, residency), hospitalization costs, length of hospital stay, insurance status, discharge status, and medical history, as well as clinical information regarding post-stroke outcomes (National Institute of Health Stroke Scale) of patients who have suffered an AIS and have received intravenous rtPA.

### 2.4. Costing Methodology

Direct and indirect costs were estimated for first-ever AIS patients within the first year after stroke, based on a set of assumptions (Table 1).

Medical costs incurred during the acute phase were estimated using a top-down approach, while medical costs from the chronic phase were computed using a bottom-up approach. Productivity costs were calculated as follows: costs for disability were estimated using a bottom-up methodology, while mortality-related costs and medical leave were estimated using a top-down approach. Costs for informal care were computed using a proportional estimate, and costs due to pensions (state pension and severe handicap subsidy) were computed using a top-down approach.

Acute care was defined as in-hospital services, such as ER services, endovascular procedures, neurology inpatient ward services, and hospital subsidies (overhead, staff, and other expenses). Costs per patient during initial inpatient hospitalization at CEH were extracted from administrative information available at discharge. Healthcare expenditure in the chronic phase was estimated using a bottom-up methodology. Based on our interviews with key stakeholders, we assumed that, on average, an AIS survivor requires an initial neurology consultation, followed by two check-ups during the first year after stroke. The framework contract for providing medical services at the national level stipulates that a patient has access to one initial rehabilitation consultation, two check-ups, and twenty-one procedures [28,29]. The cost of primary care was calculated by averaging quarterly values for per capita and per service funding, multiplied by the number of hospitalized patients (in-hospital deaths were excluded), and the number of points and consultations [30]. Hospital subsidies were computed by dividing expenses from state budget subsidies and expenses from local budget subsidies [31] to the total hospitalization days in 2019. The result was multiplied by patient sample size and the average length of stay for stroke patients. 

We calculated indirect costs using a mixed approach. Costs generated by disability were computed using a bottom-up approach. For the matched group, we added NIHSS scores corresponding percentage of the disability levels presented in an article written by Douiri et al. [32] and the values for disability weights used in the Global Burden of Disease Study [33]. Next, we calculated the cost of mortality for members of the active population who have died before hospital discharge. The number of patients from the active population who died during initial hospital admission (*n* = 7) was added to the GDP per capita for 2019 [34] and to the exchange rate for June 2019 for USD [35]. The cost for state pension was obtained by multiplying the number of inactive patients by the number of months and the average value for a state pension in 2019 for Cluj county [21]. The age threshold for the inactive population was calculated based on gender, date of birth, and month of retirement of the active population. Costs generated by patients who have died before hospital discharge were used in computing the cost of mortality for first-ever AIS patients. The cost of subsidies for disabled stroke survivors was calculated by multiplying the number of active patients from the population with the average value for social compensation [11,21]. Informal care was estimated based on the ratio between informal care and productivity from the “European Cardiovascular Disease Statistics 2017” report [22]. Descriptive statistics and visualizations were performed using Tableau Desktop 2020 software. Additional analyses were performed using Microsoft Excel. Costs are expressed in RON and EURO (€) at an exchange rate of 4.84 RON.

## 3. Results

Our analysis identified 690 first-ever AIS patients discharged alive from inpatient care on CEH neurology wards in 2019, of which 67.39% of patients were coded with I63.3 and 32.61% with G81. An additional 11 patients were transferred to neighboring Mures county for mechanical thrombectomy, and 11.76% (*n* = 92) died during hospitalization. Fifty-nine percent (*n* = 410) of patients resided in urban areas and 52% (*n* = 359) of patients were women (Table 2). The average age among men was 70 years, while the average age among women was 74 years. Out of 690 stroke survivors, 152 patients received IV thrombolytic treatment (22%).

**Figure 1:**
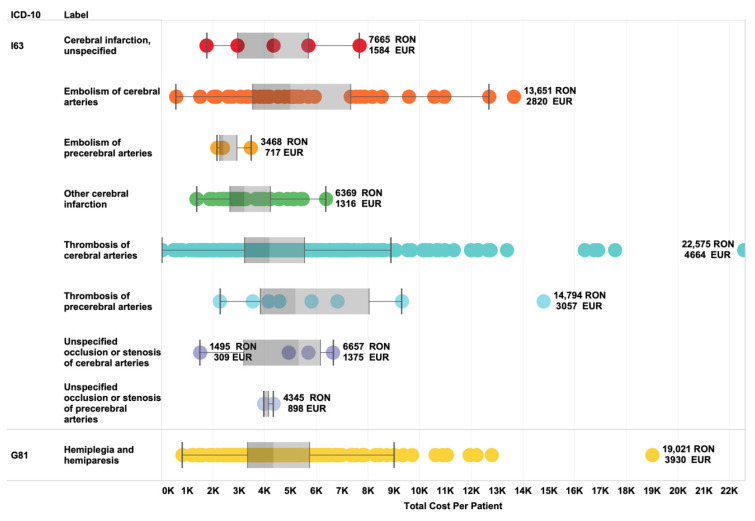
Distribution of average total cost per patient across ICD-10 diagnosis categories.

In our study cohort, 142 first-ever ischemic stroke patients were under the average retirement threshold of 62.5 years of age. The remaining 135 patients lost in 2019 a total of 1080 working days due to their hospitalization, not considering medical leave after discharge. The total cost per patient of initial inpatient admission in neurology wards varies considerably across ICD-10 diagnoses (minimum = 2675 RON–embolism of cerebral arteries; maximum = 7095 RON–thrombosis of precerebral arteries; mean = 4726 RON) (Figure 1). The mean LOS was 8.3 days.

**Figure 2:**
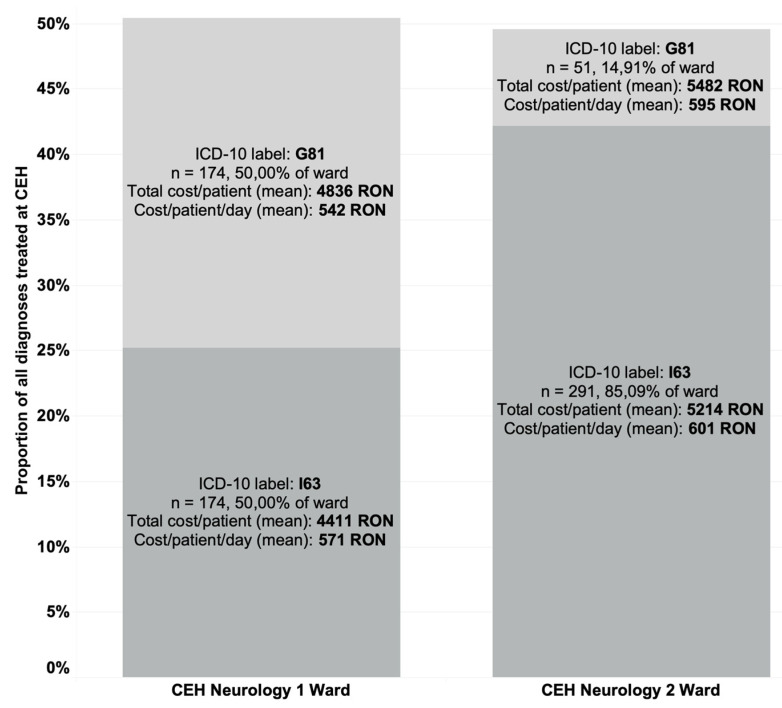
The difference in total cost and codification across G81 and I63 categories and CEH neurology wards.

The difference between average total costs and coding distribution across wards can be observed in Figure 2.

The average direct medical cost in 2019 per first-ever AIS patients in their first year after stroke onset was EUR 4227.23 (RON 20,459.84), comprising costs with emergency room services, endovascular procedures (if applicable) and neurology inpatient admission (61.12% of the direct medical costs), hospital subsidies (26.8% of direct medical costs), outpatient neurology care (0.95% of direct medical costs), outpatient rehabilitation (8.37% of direct medical costs), and primary care (2.72% of direct medical costs). The indirect cost was, on average, RON 4837.99 (EUR 999.58) per patient, dominated by informal care (45.70%) and disability-related (37.25%) costs (Table 3).

**Figure 3:**
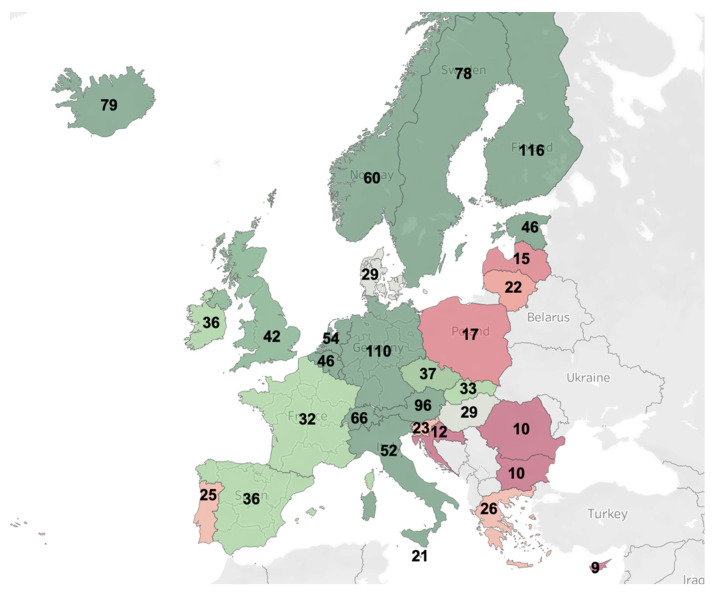
Secondary data analysis of stroke-related healthcare expenditure as compared to the population in displayed countries. Numbers represent cost per capita in Euros for the reference year 2017.

To highlight differences in stroke care expenditure across the European Union, we computed a map of healthcare costs associated with stroke per capita, as extracted from a population-based cost analysis of economic burden, visualized in conjunction with country populations as reported by the World Bank in 2017 [36,37] (Figure 3).

## 4. Discussion

Stroke was the second cause of mortality in Romania in 2016 [15]. Due to its preventable nature and high economic and social toll, it is a public health problem of great importance at the national level [38]. Although studies have been carried out to estimate the economic toll among Romanian stroke survivors [17,18], their focus has been on in-hospital costs. To our knowledge, our retrospective study is the first to calculate the burden of stroke from a societal perspective in Romania.

As seen in Table 2, first-ever AIS cases are more prevalent in older age groups, in line with findings presented in the literature [39,40]. Despite women having a lower age-adjusted risk for stroke overall, most AIS patients, over 80%, presenting at CEH were women. This could be explained by the fact that women have a significantly higher life expectancy than men in Romania, hence suffering from multiple comorbidities, and have additional risk factors compared to men [41]. 

Based on our costing analysis, costs generated by first-ever ischemic stroke patients within the first year are dominated by direct costs (81.55%). The average in-hospital cost for women was RON 5233.15 (EUR 1081.23) and RON 5519.84 (EUR 1140.46) for men. For all patients, the highest inpatient costs were attributable to hospitalization and laboratory costs. As part of the case selection process, identified first-ever AIS cases with a primary diagnosis were coded with G81 (instead of the corresponding I63 codes), showcasing systemic issues with hospital funding in Romania. Based on our key informant interviews, secondary and tertiary rehabilitation needs are grossly underserved, and some services are lacking almost entirely (e.g., speech therapy). As portrayed in Figure 3, Romania has a lower estimated stroke-related healthcare expenditure than other European Union members. This example of inadequate financial resource allocation stems from the overarching issue of low overall expenditure for health in general for Romania (5.5% vs. 9.9 EU average in 2018) [42]. This historically perpetuated deficiency has a profound impact on patient access and standards of care, leading to potential restrictions in applying clinical guideline recommendations. In the wake of the COVID-19 pandemic, there have been important barriers in access to healthcare in Romania, particularly for chronic patients. More worryingly, discharges for acute services have also declined, compounding the effects of chronic underfunding for healthcare at the national level [43]. 

Precise comparison of results with cost of stroke studies from other countries is difficult due to differences in study population (first-ever events [44] and recurrent strokes are included [45]), stroke type [46], medical coding [47], demographic characteristics [6], perspective [47], costing methodology [48], timeline (studies have reported costs for 1 [46], for 5 years [49] or for lifetime [50]), design (retrospective [51] or prospective [52]), data sources (primary [53] or secondary [54]), costing items [46,55], and healthcare expenditure as a percentage of the GDP in each country [19]. As expected, findings indicate lower figures as compared to other high-income countries [46,47,56] and higher expenditure than developing countries [53].

The previous cost-of-stroke studies conducted in Romania focused exclusively on in-hospital costs. Uivarosan et al. (2020) conducted their study in a thrombolysis center and followed AIS patients for two years [17], yielding lower comparable costs. The results of our costing exercise are similar to those reported by Lorenzovici et al. (2020), where hospitalization and laboratory tests are the highest inpatient cost, drivers. This study used hospital financial controlling data to measure in-hospital costs generated by stroke patients from six hospitals (first-ever and recurrent cases), classified according to I60.0–I66.9 (ICD-10). Our average figures for direct medical cost lie between “Clinical Hospital 2” (EUR 1230.27) and “City Hospital 2” (EUR 1011.55), as reported in the study mentioned above [18].

Limitations of our approach include several assumptions described in our methodology section, setting (a single-center study that focuses on a pre-defined catchment area), exclusion of patients undergoing mechanical thrombectomy (which are transferred to the nearest county that provides it), lack of a longitudinal component focusing on the quality of life of patients, which would add a more detailed view of indirect costs incurred by stroke survivors. Our study also fails to include transportation costs for patients residing outside of Cluj-Napoca and patients admitted to rehabilitation wards through interhospital transfer or on-demand. Informal care provided by a family member or by paid caregivers is not included. This is a gap in our analysis, since studies have shown that even though patients had access to rehabilitation, they still need daily help after stroke onset [57]. Finally, this study does not consider out-of-pocket expenditure in the private sector (roughly 20% of financing at the national level) and informal payments. Based on these limitations, we conclude that the amount of indirect cost is likely to have been underestimated. Our study has several methodological strengths, including using all first-ever AIS patients hospitalized in the timespan of one year in the selected center and calculating stroke-related costs from the societal perspective for one year, the recommended time window for the cost of illness studies [20]. Our work also provides insight into the incidence of first-ever AIS cases in Cluj county in 2019, the equivalent of 164 cases per 10,000 inhabitants.

## 5. Conclusions

We conclude that new information provided by this costing exercise, which focused on first-ever AIS cases, may provide insight regarding the economic burden of AIS, informing local and central government efforts to improve standards of care. Local and central authorities from Romania must urgently take steps to improve the status quo by ensuring appropriate financial and human resource allocation for stroke care, investing in stroke units, and building robust health information systems to inform decision-making.

In the future, national-level costing exercises should include all stroke subtypes in an interwoven analysis, as patients diagnosed with hemorrhagic stroke consume higher resources [58] and those suffering from transient ischemic attacks are at increased risk of subsequent stroke [59]. As the Romanian healthcare system continues its reform, the societal perspective cost of illness studies targeting ischemic stroke and other prevalent non-communicable diseases must become pillars of evidence-based financing policies and health system performance evaluation. In light of COVID-19, access of stroke patients to appropriate acute and chronic level care must be closely monitored to ensure that patient pathway issues identified by our study are not compounded by additional pressure placed by the ongoing pandemic on health infrastructure and budgets.

## Figures and Tables

**Table 1:** Assumptions made when estimating costs.

Cost Item	Assumptions
**Outpatient chronic services** (Neurology)	AIS survivors receive an initial neurology consultation, followed by two check-ups during the first year after stroke.
**Outpatient chronic services** (Rehabilitation)	AIS survivors receive one initial rehabilitation consultation, two check-ups, and twenty-one rehabilitation procedures.
**Primary care**	For stroke patients, primary care interactions are mostly stroke-related.
**Productivity loss**	
*Disability*	Only moderate-severe patients develop cognitive decline.
*Mortality*	Only deaths during hospitalization are considered.
*Medical leave*	The average retirement threshold is 62.5 years of age. Hospitalization days have been considered as medical leave days. Follow-up medical leave (outpatient setting) is not taken into account. Patients who have died during hospitalization are omitted.
*Social services*	Moderate, severe cases are eligible for disability compensation.

**Table 2:** Gender, length of stay (LOS) and cost of first-ever AIS patients treated at CEH in 2019 by age groups.

Age Group	Sample (*n*)	Gender (%Female)		Average LOS	Average Cost per Day (RON) (€)	
30	5	80%		6.4	608.8	125.78
40	29	55%		6.9	677.8	140.04
50	59	32%		7.8	611.2	126.28
60	181	41%		8.2	605	125
70	236	53%		8.8	564.8	116.69
80	161	65%		9.3	547	113.01
90	19	84%		10.3	537.5	111.05

**Table 3:** Direct medical, direct non-medical, and indirect costs mean cost in the first year after AIS onset.

	Per Patient (RON)	Per Patient (€)	Per Cohort (RON)	Per Cohort (€)	Methodology
**Direct medical cost**					
*Acute care*					
ER, endovascular procedures, neurology wards	4840.63	1000.13	3,340,034.70	690,089.70	Top-down
Hospital subsidies (overhead, staff, and other expenses)	2123.27	438.69	1,465,056.30	302,696.10	Top-down
*Chronic care*						
Outpatient neurology	74.88	15.47	51,667.20	10,674.30	Bottom-up
Outpatient rehabilitation	662.88	136.96	457,387.20	94502.4	Bottom-up
Primary care	215.4	44.5	148,626.00	30,705.00	Bottom-up
*Total direct medical cost*	*7917.06*	*1635.75*	*5,462,771.40*	*1,128,671.77*	
**Direct non-medical cost**					
*Pensions (social services)*					
State pensions	12,538.17	2590.53	8,651,337.30	1,787,465.70	Top-down
Severe disability subsidies	4,61	0.95	3180.90	655.5	Top-down
*Total direct non-medical cost*	*12,542.78*	*2591.48*	*8,654,518.20*	*1,788,121.20*	
**Total direct cost**	**20,459.84**	**4227.23**	**14,117,289.60**	**2,916,788.70**	
**Indirect cost**					
*Productivity loss*					
Disability-related	1802.27	372.37	1,243,566.3	256,935.30	Bottom-up
Mortality-related	548.52	113.33	378,478.80	78,197.70	Top-down
Medical leave	275.97	57.02	190,419.30	39,343.80	Top-down
Informal care	2211.23	456.87	1,525,748.70	315,240.30	Proportional estimate
**Total indirect cost**	**4837.99**	**999.58**	**3,338,213.10**	**689,710.20**	
**Total cost**	**25,297.83**	**5226.82**	**17,455,502.70**	**3,606,505.80**	

## Data Availability

Replication data for this study is available in the Harvard Dataverse: (doi:10.7910/DVN/OYXE83).
